# Autophagy and Biomaterials: A Brief Overview of the Impact of Autophagy in Biomaterial Applications

**DOI:** 10.3390/pharmaceutics15092284

**Published:** 2023-09-05

**Authors:** Leila Pirmoradi, Shahla Shojaei, Saeid Ghavami, Atefeh Zarepour, Ali Zarrabi

**Affiliations:** 1 Department of Medical Physiology and Pharmacology, Faculty of Medicine, Kurdistan University of Medical Sciences, Sanandaj 66177-13446, Iran; lpirmoradi@gmail.com; 2 Department of Human Anatomy and Cell Science, Max Rady College of Medicine, University of Manitoba, Winnipeg, MB R3E 0V9, Canada; shojaeis2019@gmail.com; 3 Academy of Silesia, Faculty of Medicine, Rolna 43, 40-555 Katowice, Poland; 4 Research Institute of Oncology and Hematology, Cancer Care Manitoba-University of Manitoba, Winnipeg, MB R3E 0V9, Canada; 5 Children Hospital Research Institute of Manitoba, University of Manitoba, Winnipeg, MB R3E 0V9, Canada; 6 Department of Biomedical Engineering, Faculty of Engineering & Natural Sciences, Istinye University, Istanbul 34396, Türkiye; atefeh.zarepour@gmail.com

**Keywords:** biomaterials, autophagy, osteogenesis, angiogenesis, neurodegenerative diseases, tumor cells

## Abstract

Macroautophagy (hereafter autophagy), a tightly regulated physiological process that obliterates dysfunctional and damaged organelles and proteins, has a crucial role when biomaterials are applied for various purposes, including diagnosis, treatment, tissue engineering, and targeted drug delivery. The unparalleled physiochemical properties of nanomaterials make them a key component of medical strategies in different areas, such as osteogenesis, angiogenesis, neurodegenerative disease treatment, and cancer therapy. The application of implants and their modulatory effects on autophagy have been known in recent years. However, more studies are necessary to clarify the interactions and all the involved mechanisms. The advantages and disadvantages of nanomaterial-mediated autophagy need serious attention in both the biological and bioengineering fields. In this mini-review, the role of autophagy after biomaterial exploitation and the possible related mechanisms are explored.

## 1. Introduction

### 1.1. Autophagy

**Figure 1:**
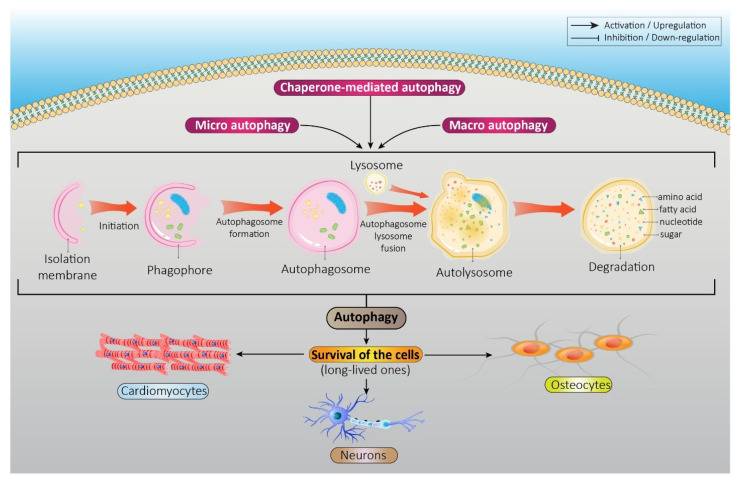
Different types of autophagy: Chaperone-mediated autophagy, microautophagy, and macroautophagy are distinct forms of autophagy, all serving the ultimate purpose of delivering cellular cargo to the lysosome for degradation. Chaperone-mediated autophagy involves the recognition of specific proteins by cytosolic chaperones, which facilitate their translocation across the lysosomal membrane. Microautophagy occurs when the lysosome directly engulfs portions of the cytoplasmic contents through the invagination or protrusion of its membrane. In contrast, macroautophagy involves the sequestration of cargo within double-membrane vesicles called autophagosomes, which then fuse with lysosomes for degradation. Despite their different mechanisms, all three types of autophagy contribute to maintaining cellular homeostasis by removing unwanted or damaged components, thereby enabling recycling and nutrient replenishment.

Autophagy is a recycling process of damaged organelles, macromolecules, and nutritious proteins that basically protect cells during starvation or stress conditions by digesting cytoplasmic materials into metabolites due to energy production [1,2,3]. Indeed, it is a crucial mechanism for the survival of the cells, especially long-lived ones, including osteocytes, neurons, and cardiomyocytes. Autophagy has an important role in homeostasis under physiological conditions and acts as a double-edged sword during stress. Under stress conditions, autophagy acts as a cytoprotective mechanism or exerts adverse effects through inadequate or excessive activation. Therefore, regulated autophagy in basal and induced levels determines the autophagy impact on cells. The induction of autophagy occurs during development and in response to various stressors, including starvation, hypoxia, metabolic or oxidative stress, and DNA damage [4]. Autophagy dysfunction plays a significant role in pathologies such as infections, myopathies, cancer, aging, and neurodegenerative and metabolic diseases [5,6,7]. The three types of autophagy that finally deliver cargo to lysosomes are macroautophagy, chaperone-mediated autophagy, and microautophagy [8] (Figure 1). In microautophagy, lysosomes engulf small protein complexes through the invagination of the lysosomal membrane [9]. Chaperone-mediated autophagy is a process in which misfolded proteins are conveyed to lysosomes by chaperone proteins and subsequently broken down by lysosomal hydrolases [10]. Macroautophagy is the main form of autophagy (henceforth autophagy) in which defective organelles are degraded. In this process, the target is surrounded by a double-membrane vesicle and transported intracellularly to fuse with lysosomes, leading to degradation [11].

The autophagic process comprises four steps: initiation, nucleation, maturation, and degradation. Several autophagy-related genes (ATGs) are involved in this process, and different ATG proteins regulate these four steps [12].

Autophagy is regulated through a network of signaling pathways involving key regulatory proteins, such as serine–threonine protein kinase, a mechanistic target of rapamycin complex 1 (mTORC1) [13], AMP-activated protein kinase (AMPK), and the class III phosphatidylinositol 3-kinase complex (PI3KC3) [12]. Low levels of amino acids, growth hormones, and energy are the main triggers of autophagy induction that have been studied so far [4]. Autophagy is inhibited by mTOR [14], which inhibits the initiation of autophagy upon receiving growth factors and nutrients. Conversely, under stress conditions or when nutrients are limited, AMPK is activated, leading to mTORC1 inhibition and autophagy induction. The PI3KC3 complex, containing Beclin-1, is essential for autophagosome formation. It generates phosphatidylinositol 3-phosphate (PI3P), which recruits other autophagy-related proteins to initiate autophagosome biogenesis [12].

Autophagy is initiated via the activation of the ULK complex, which is necessary for the recruitment of downstream-related proteins to boost phagophore nucleation. The only transmembrane core autophagy machinery protein, Atg9, is a target of ULK-mediated phosphorylation activation, which is responsible for membrane trafficking for nucleation and the expansion of the phagophore at the onset of autophagy [15,16].

The process of phagophore formation, a critical step in autophagy, relies on the activity of the class III PI3K complex (PI3KC3). There are two types of PI3KC3 complexes, I and II. Complex I consists of six subunits: Beclin-1, Vps15, Atg14, AMBRA1, NRBF2, and hVPS34 [17,18]. In normal conditions, AMBRA1 stabilizes complex I on the cytoskeleton by interacting with Beclin-1. However, when autophagy induction occurs, the mTOR-mediated inhibitory phosphorylation of AMBRA1 is removed, leading to interactions with TRAF6, promoting UKL1 and Beclin-1 ubiquitination, and facilitating ULK complex kinase activity. ULK complex activation triggers the translocation of PI3KC3 complex I towards the ER and then initiates nucleation [19]. Furthermore, Beclin-1 is phosphorylated by ULK, activating the hVPS34 complex, which catalyzes the production of phosphatidylinositol 3 phosphates (PI3P), providing docking sites for PI3P-binding proteins DFCP1 and WIPI, thus facilitating Atg16L. Atg14 and NRBF2 are also regulated by mTOR. Atg14 is essential for PI3KC3 complex I with autophagosome attachment [19].

Phagophore expansion is the next step that involves Atg12 and microtubule-associated protein light chain 3 (LC3). Atg12 is activated by Atg7 and binds to Atg5, forming a complex that, along with Atg16L, regulates the bending of the phagophore membrane [20]. The other conjugation system involves the lipidation of LC3 and its homologs, resulting in the formation of LC3-I and then LC3-II through Atg7 and Atg3 conjugation with phosphatidylethanolamine (PE) [21]. The Atg12–Atg5–Atg16L complex also participates in the LC3-II–PE conjugation process, facilitated by WIPI2, and is transiently associated with the phagophore membrane [4].

During the cargo targeting process, autophagy can be selective or non-selective. In the non-selective autophagic process, protein aggregates and other cytoplasmic organelles, such as mitochondria, endoplasmic reticulum, and Golgi membranes, are delivered to the expanding phagophore for recycling. On the phagophore surface, LC3-II acts like a receptor and scavenges targets to the phagophore [22]. Selective autophagy includes mitophagy, ER-phagy, Pexophagy, Xenophagy, and Ribophagy [4].

Fusion and degradation are the final steps of autophagy flux. When the autophagosome is completed, PE-conjugated LC3-II is broken. Autophagosomes and lysosomes are dragged together with the help of cellular trafficking proteins, and the outer membrane of the autophagosome is fused to the lysosomal membrane [23,24].

Stimuli such as oxidative stress, starvation, mitochondrial toxins, and hypoxia induce autophagy through ROS generation. ROS probably induce autophagy through some different mechanisms involving Atg4, catalase, and the mitochondrial electron transport chain (mETC), leading to cell survival and cell death [25].

### 1.2. Biomaterials

Biomaterials encompass substances derived from nature or synthesized materials, falling into four main categories: metals, ceramics, polymers, and composites. These materials find applications in both diagnostics and therapeutics, such as tissue treatment, augmentation, repair, or replacement [26,27]. For instance, they could be exploited in dentistry and orthopedic operations, surgery, drug delivery, and as scaffolds in tissue regeneration to interact with biological systems [28]. They could be fabricated from organic and inorganic materials in the range of nanoscale to exhibit autophagy-modulating effects [29,30,31,32]. In this case, the physical, chemical, and biofunctional properties of nanomaterials exert modulatory effects on autophagy through various mechanisms [33,34]. Specifically, the high surface-to-volume ratio of these materials allows for the loading of substantial therapeutic compounds that can either activate or suppress autophagy. In addition, their topography, such as porous structure, can trigger autophagy by inducing cytotoxicity or regulating cellular mechanisms [35,36]. In terms of their biological impact, nanomaterials can directly influence autophagy (via upstream responses) and/or exert their effects through other biological factors. For instance, zinc oxide (ZnO) nanoparticles and silica nanoparticles (SiNPs) can induce autophagy by disrupting the activity of antioxidant enzymes, thereby increasing levels of reactive oxygen species (ROS) and inhibiting signaling pathways like PI3K/Akt/mTOR, leading to induced toxicity [37]. In the following table (Table 1) the effects of the physicochemical properties of nanomaterials on autophagy are summarized.

## 2. Biomaterials and the Role of Autophagy

### 2.1. Biomaterials, Osteogenesis, and Autophagy

Bone health relies on a fundamental process known as osteoimmunology, which involves various cellular and molecular mechanisms. Notably, autophagy stands out as a pivotal process that preserves homeostasis even under stressful conditions like starvation and hypoxia. Autophagy plays a crucial role in osteoclast differentiation and immune cell activation, effectively mitigating the detrimental impacts of oxidative stress on cells. In addition, it contributes to the maintenance of the bone marrow hematopoietic stem cell niche [40]. Osteogenesis is promoted when autophagy genes are upregulated, and inflammation is suppressed by raising the M2 polarization of macrophages [41].

**Figure 2:**
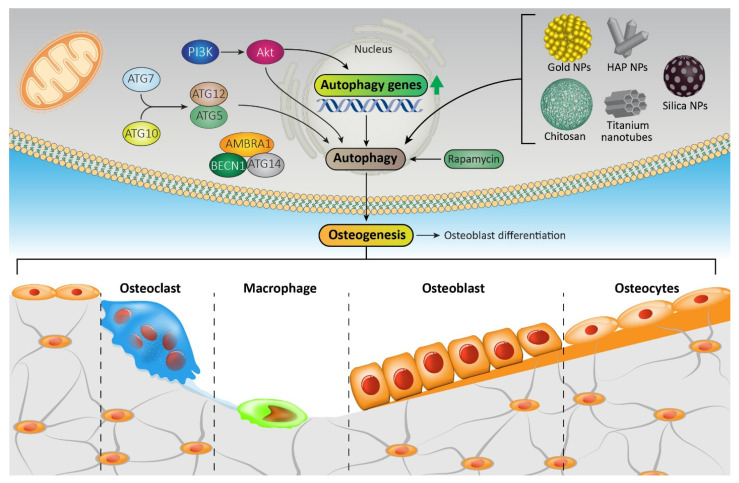
The interaction of biomaterials with biological systems in osteogenesis: The interaction between biomaterials and biological systems, including osteoclasts, macrophages, osteoblasts, and osteocytes, is pivotal in the context of osteogenesis, facilitating the process of bone formation and regeneration. Osteoclasts, specialized cells derived from macrophages, are responsible for bone resorption and remodeling. Macrophages, key players in the immune response, interact with biomaterials and contribute to the modulation of inflammation and tissue healing. Osteoblasts, responsible for bone matrix synthesis, interact with biomaterials to promote new bone formation. Osteocytes, the most abundant cells in bone tissue, play a vital role in maintaining bone health and orchestrating the communication between different cell types. The intricate interplay between biomaterials and these cellular components is essential for optimizing the performance of biomaterials in promoting osteogenesis and achieving successful bone regeneration. The arrow indicates activation.

Biomaterials, broadly categorized into metals, polymers, ceramics, and composites, interact with biological systems and play a vital role in osteogenesis (Figure 2). Silver, alumina, titanium, and gold also find extensive applications in bone regeneration [26].

Two primary concerns that have adverse effects on osseointegration are the decline in material characteristics and heightened osteoclast activity. Oxidative stress stimulates the differentiation of osteoclasts and leads to the production of glycation end products (AGEs), thereby contributing to the deterioration of material characteristics [42]. Some factors that upregulate the PPAR signaling pathway promote the differentiation of osteoclasts, leading to bone loss [43].

Biomaterials play a pivotal role in promoting bone regeneration through their involvement in the autophagic process. Bone regeneration induced by biomaterials is influenced by biomaterial–cell interface and surface topography modification to achieve proper cell growth and differentiation [26]. In this regard, immune system interaction with biomaterials improves the bone tissue regeneration performance of implants. The impacts of differently sized nanoporous anodic alumina on macrophage responses are particularly significant, leading to changes in the differentiation of bone marrow stromal cells. The nanopore structure and the pore size intricately orchestrate the autophagy pathway through activation of LC3A/B, Beclin-1, Atg3, Atg7, and P62, consequently influencing osteoclastic activity, the release of osteogenic factors, and the inflammatory response. Indeed, the knowledge of how immune cells interact with nanotopography-mediated osteogenesis holds the potential to drive the development of advanced nanobiomaterials for various medical applications [44,45].

Silicon-based materials, specifically in polymer forms, exhibit proangiogenic effects and find extensive use in regenerative medicine. These materials serve as scaffolds in bone tissue regeneration. Orthosilicic acid, a soluble form of silicon, boosts the differentiation and mineralization of osteoblasts through the induction of the autophagic process [46]. It has also been reported that bioactive silica nanoparticles (NPs) stimulate osteoblasts, inhibit osteoclasts in vitro, and increase bone mineral density (BMD) in vivo through the involvement of autophagy [47,48]. Intriguingly, when it comes to stimulating autophagy for osteoblast mineralization, the nanoparticle size (50 nm) proves more crucial than the specific type of nanoparticle [47,49]. A similar study showed that 45 nm gold nanoparticles (AuNPs) exhibit the most stimulatory effects on autophagy and osteogenesis [50]. Chitosan, a polysaccharide copolymer derived from crustacean shells, stands out for its better differentiation potential of MSCs into mesenchymal lineages like osteoblasts [26]. When chitosan film forms three-dimensional spheres for culturing MSCs, it increases the expression of Oct4, Nanog, and Sox2, consequently promoting osteogenic differentiation potential [51].

Among metals, titanium stands out due to its biocompatibility and mechanical properties, making it highly suitable for various orthopedic and dentistry usage [52,53]. The modulatory impact of bone morphogenetic protein 2 (BMP2) on macrophages presents an opportunity for functionalizing scaffolds. This can be achieved through the integration of exosomes from BMP2-stimulated macrophages, effectively preventing ectopic bone formation and minimizing adverse effects [54]. Wei et al. harnessed BMP2-/macrophage-derived exosomes to improve the biofunctionality of titanium nanotube implants for osteogenesis. They showed that titanium nanotubes incorporated with BMP2-/macrophage-derived exosomes exert osteogenic effects through autophagy activation [55]. Kaluđerović et al. reported that the autophagic-dependent PI3/Akt signaling pathway is essential for osteoblast differentiation on rough topographic titanium-based surfaces. The presence of mature osteoblasts and small granule cells forming cell clusters, ultimately crucial for bone nodule formation and mineralization, is heavily reliant on autophagy activation [56]. The surface elasticity modulus of titanium (Ti) implants plays a crucial role in the regulation of MSCs. In this context, spherical silica nanoparticles (SSNs) have been designed to improve osteointegration between the implant surface and bone tissue. Ti-SSNs have been shown to stimulate a higher level of autophagosome formation and mineralization [57].

The nanotube structure enhances mTOR-independent autophagy in osteoblasts compared with flat surfaces. Non-topographic surfaces stimulate reversible and memorable autophagy through the stretching of the cell membrane, leading to cell differentiation [58]. Nanotopography has a stronger effect on autophagy activation and osteogenesis than smooth surfaces. This effect has also been reported when titanium implants with rough topographical surfaces are used that induce elevated nuclear β-catenin, osteogenic transcription factors, and lower levels of cytoplasmic YAP (Yes-associated protein) in MC3T3-E1 cells [59].

Wang et al. designed a Sr-doped micro/nano rough titanium implant surface using hydrothermal treatment (SLA+Sr), revealing that the in vitro SLA+Sr surface increases the differentiation of bone-marrow-derived mesenchymal stem cells (BMSCs) through the autophagic process [60]. Interestingly, substrate stiffness stimulates osteogenic responses in vascular SMCs as well. It has been observed that cells on the substrates with intermediate stiffness (0.909 MPa) exhibit the highest extent of osteogenesis through the involvement of transforming growth factor-β1 and autophagy [61].

Regarding the implant surface modified with nanotubes for bone tissue engineering, Chernozem et al. prepared hybrid composites using Ti−xNb alloys and oxide nanotubes (NTs) as a platform for improved adhesion of hMSCs. The hybrid composites included β-alloy Ti−xNb and oxide nanotubes under electrochemical anodization at different voltages (30 V and 60 V) and Nb contents (5, 25, and 50 wt %). Scanning electron microscopy revealed the formation of vertically aligned nanotubular structures on Ti−Nb substrates, with distinct impacts on Nb content depending on the lengths of the NTs. Both anodization voltage and the Nb content exerted influence over Young’s modulus and the stiffness of NT arrays. Indeed, the morphology of NTs plays a role in the determination of hardness and Young’s modulus, and lower anodization voltage results in highly dense morphology. According to the results of this study, optimal hMSC adhesion was achieved when utilizing NTs with an inner diameter of approximately 50 nm and an anodization voltage of 30 V. The same conditions also prompted the proliferation of hMSCs [62].

Gold nanoparticles (AuNPs) exhibit osteogenic differentiation effects on periodontal ligament stem cells (PDLSCs). AuNPs increase osteogenesis in PDLSC sheets by activating autophagy, via microtubule-associated protein light chain 3 upregulation and sequestosome 1/p62 downregulation [63]. Li et al. assessed the role of autophagy in the differentiation and mineralization of human dental pulp stem cells induced by fluorapatite (FA) crystal-coated electrospun polycaprolactone (PCL). In their study, several autophagy-related genes and proteins were altered during the differentiation of human adipose-derived stem cells (ASCs), and when autophagy was inhibited, the osteogenic differentiation and mineralization of ASCs were also inhibited, as observed in the three-dimensional model [64].

Pulp regeneration requires tissue repletion achieved through adequate vascularization, neuron formation, and dentin deposition. The process that provides these necessary functions is autophagy, which is essential for angiogenesis, neural differentiation, and osteogenesis. In vitro studies revealed that the migration and regeneration of stromal-cell-derived factor-1α (SDF-1α)-mediated dental pulp stem cells (DPSCs) are mediated by autophagy. An in situ pulp revascularization model showed that the de novo ingrowth of pulp-like tissues in pulpectomies of mature dog teeth was improved with SDF-1α-loaded silk fibroin scaffolds related to the autophagic expressions of LC3 and Atg5. It has also been shown that after the ectopic transplantation of tooth fragment/silk fibroin scaffold with DPSCs in mice, pulp-like tissues with vascularity, desirable fibrous matrix formation, and young dentin deposition were obtained in SDF-1α-loaded samples associated with autophagy [65]. Osteoporosis, a significant bone disease impacting bone homeostasis, was investigated by Zhang et al. They designed a biomaterial, strontium (Sr)-doped 45S5 bioglass (Sr/45S5), and utilized ovariectomy bone-marrow-derived mesenchymal stem cells (OVX-BMSCs) for their cell culture model. Their results showed that the Sr-induced osteogenic differentiation of OVX-BMSCs was related to autophagy modulation in a time-dependent manner and the AKT/mTOR signaling pathway. The in vivo models of femoral condyle defects in OVX rats also showed that Sr10/45S5 granules improved bone regeneration. It seems that an early improvement in autophagy and the late activation of the Akt/mTOR signaling pathway can promote the osteogenic differentiation of OVX-BMSCs and bone regeneration in the osteoporotic defects of bone using Sr doping [66].

Hydroxyapatite scaffolds (HASs) are widely used as compatible materials for bone substitution. In this context, fabricated HAS with a 25–30 µm groove structure (HAS-G) increases osteogenesis through macrophage-induced immune microenvironment modulation. Compared with HAS, HAS-G can inhibit ROS production, mitophagy-induced ROS elevation, and ATP synthesis [67].

Certain drugs exert influence over both autophagy and cellular differentiation processes. Rapamycin (RAPA) was assessed regarding the differentiation of maxillary sinus membrane stem cells (MSMSCs). In this study, Zhang et al. showed that RAPA increases osteogenic differentiation with autophagy involvement [66]. Metformin is another effective drug in this regard. While polydopamine-templated hydroxyapatite (tHA) increases ROS production in higher concentrations, it can boost osteogenesis. Yang et al. illustrated that when human periodontal ligament stem cells (hPDLSCs) are exposed to tHA and metformin, ROS production is limited. According to this study, autophagy and AMPK/mTOR signaling pathways are also involved, resulting in osteogenesis [68].

Fluorapatite (FA)-modified polycaprolactone (PCL) nanofiber represents a promising scaffold for odontogenic–osteogenic tissue engineering, as it triggers stem cell differentiation and mineralization. This trend is intricately modulated via the hedgehog, insulin, and Wnt signaling pathways and mediated via the autophagic process [69].

The morphology of nanoparticles is another factor that has a positive impact on osteogenesis. Yang et al. used a modified liquid–solution–solid (LSS) method to synthesize hydroxyapatite NPs with suitable morphologies. These NPs had differential effects on stem cells, reflected in particle uptake, autophagy activation, and osteogenic differentiation. They showed that, compared with other NPs, spherical particles strongly induce autophagy and bone formation [70].

Autophagy has a role in particle-related diseases, caused by different factors such as particles released from artificial joint friction, implant materials, scaffolds used in tissue engineering, and materials for drug delivery. Titanium (Ti) particle-induced particle disease that activates autophagy marker LC3 was modulated using nanosized aluminum in MG-63 cells in a mouse calvarial osteolysis model. It could prevent autophagy and decrease osteolysis induced by Ti particles [71]. Different effects of nanomaterials used in osteogenesis on autophagy are summarized in Table 2.

### 2.2. Nanomaterials and Autophagy in Tumor Cells

Common therapeutic approaches in cancer treatment include surgery, chemotherapy, radiotherapy, and immunotherapy. The development of resistance is the main problem in the treatment of patients who are under chemotherapy [72]. The interplay between cancer and autophagy is intricate. While autophagy is related to chemoresistance [73] and necessary in the maintenance of cancer stem cells, it inhibits cancerous cell growth. Indeed, it has the opposite effect in new and well-established tumors. It suppresses tumor cells in early stages and conversely maintains cancer cells in established tumors, leading to tumor progression [74]. Autophagy, on the one hand, increases the tolerance of tumor cells to undesirable conditions such as hypoxia and acidosis, and on the other hand, it omits dysfunctional or destructed cellular organelles, especially during tumorigenesis, leading to tumor regression [75]. Therefore, both the induction and blockade of autophagy can be exploited in cancer therapies.

Nanomaterials can induce autophagy in cancer cells, which is influenced by their size, shape, surface quality, and composition. These properties affect autophagy via intracellular oxidative stress induction or alteration in gene/protein expression [75]. Nanoparticles stimulate the production of high levels of reactive oxygen species (ROS) in tumor cells, resulting in autophagy [75,76,77]. It is also illustrated that reactive nitrogen species stimulate autophagy and non-apoptotic cell death. Pt-coated Au nanoparticle contributes to the production of nitric oxide and finally NO-dependent mitochondrial dysfunction and autophagy. Since this type of nanoparticle cell death differs from the effects of conventional therapeutic methods such as ROS-inducing cisplatin, it presents a promising alternative for cancer treatment [78]. Various types of nanoparticles, including TiO_2_, ceria, iron oxide, rare earth oxides, and carbon nanotubes, have been shown to induce autophagy in cancer cells [30].

It seems that autophagy manipulation can assist conventional treatments for highly effective anticancer therapies. The achievement of potent cytotoxicity, a pivotal factor in cancer therapy, often necessitates administering high doses of antitumor drugs, like nelfinavir (NFV), which inherently limits their therapeutic potential. Fortunately, using a nanoparticle delivery system has addressed this challenge. NFV-loaded PLGA nanoparticles (NPs) induce ER stress marker ATF3, cleave PARPs, block autophagy (LC3BII upregulation), and finally cause cell death [79]. Similarly, titanium dioxide (TiO_2_) nanoparticles (NPs) enhance chemotherapy response to cisplatin in murine melanoma models by mediating the autophagy mechanism [80]. Notably, TiO_2_ has special super-photocatalytic properties that enable the eradication of tumor cells upon irradiation [30].

Chirality has an important role in autophagy. Chirality-dependent autophagy has been reported with D-type dendrimers considered dominant, as they have a stronger effect than L-type in the formation of autophagosomes and autolysosomes [75]. In the context of cancer, chiral zinc-aspartate nanofibers (L/D-(Zn-ASP) NFs) synthesized by Xin et al. have demonstrated compelling effects. These nanofibers exhibit a binding affinity to eHSP90 present in cancer cells, resulting in a reduction in gelatinase levels and the downregulation of nuclear factor-kappa B (NF-κB) signaling. Consequently, this cascade of events suppresses autophagy and effectively inhibits cancer cell proliferation, migration, and invasion [81].

Nanoparticle-based gene therapy stands out as a promising area in cancer, offering the potential to inhibit autophagy. Cationic polymers that deliver plasmid DNA (pDNA) encoding TRAIL (tumor necrosis factor-related apoptosis-inducing ligand) are able to inhibit autophagy and strongly affect tumor cells. Wang et al. reported that the copolymerization of methacryloyl chloroquine (MACQ) with 2-(dimethylamino) ethyl methacrylate (DMAEMA) increases transfection and improves autophagy capability, thus inducing apoptosis via TRAIL induction in cancer cells [82].

Using polymeric nanoparticles for the delivery of mRNA has demonstrated the ability to induce the expression of gene-encoding phosphatase and tensin homolog deleted on chromosome 10 (PTEN) in PTEN-mutated melanoma cells and PTEN-null prostate cancer cells, leading to autophagy and cell-death-associated immune activation. PTEN mRNA nanoparticles promote the CD8+ T-cell infiltration of the tumor tissue and increase the expression of proinflammatory cytokines that finally reverse the immunosuppressive tumor microenvironment [83].

In addition to their diagnostic potential, superparamagnetic iron oxide nanoparticles (SPIONs) emerge as promising biocompatible, non-cationic, and non-toxic tools for gene therapy, and they have applications in breast cancer treatment. In vitro and in vivo studies showed that functionalized SPIONs delivered an effective amount of microRNA to HER2-positive breast cancer cell lines in a xenograft nude mice model of breast cancer and thus inhibited autophagy [84].

**Figure 3:**
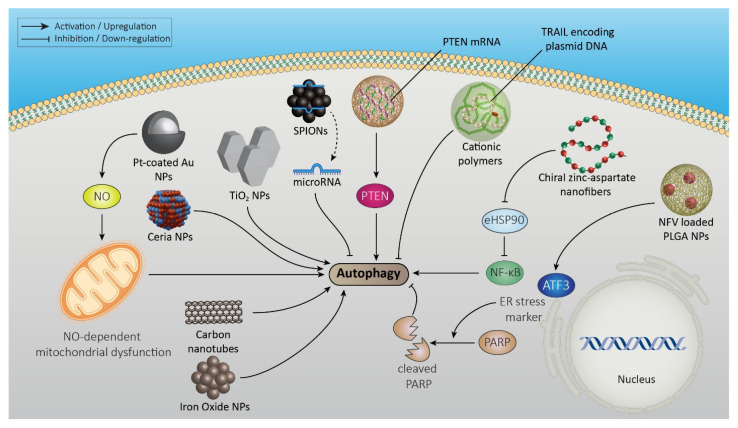
Nanomaterials can induce autophagy in cancer cells through various mechanisms depending on their size, shape, surface quality, and composition. These nanoparticles stimulate reactive oxygen species and nitrogen species, leading to autophagy and non-apoptotic cell death. Nanomaterials like TiO_2_, ceria, iron oxide, rare earth oxides, and carbon nanotubes can manipulate autophagy, improving drug delivery and overcoming resistance. Additionally, nanoparticle-based gene therapies can inhibit autophagy and induce apoptosis in cancer cells.

The utilization of chitosan nanoparticles (NPs) promotes the intracellular distribution of NPs, a phenomenon that is underscored by the enhanced transfection efficiency of genes in vitro, as elucidated in a study by Zheng et al. Notably, in vivo studies have showcased the ability of autophagy inhibitors to significantly suppress tumor growth [85]. The diverse mechanisms through which various nanomaterials impact autophagy in cancer cells are visually illustrated in Figure 3. We have concisely summarized the effects of nanomaterial features on autophagy-associated alterations in cancer cells in Table 3.

### 2.3. Biomaterials, Drug Delivery, and Autophagy

Autophagy has a profound impact on the intracellular pharmacokinetics of nanomedicine, encompassing processes like the absorption, distribution, excretion, and metabolism of these therapeutic agents, ultimately influencing their therapeutic effectiveness. The inhibition of autophagy enhances the delivery of diagnostic and therapeutic agents [89]. Nanoparticles induce autophagy in cancer cells and limit the effect of nanomedicine. Zhang et al. showed that the coadministration of 3-methyladenine (3-MA) and chloroquine (CQ) as autophagy inhibitors improves the therapeutic effects of the nanoparticles [90]. The influence of autophagy extends to one-dimensional (1D) nanostructured materials employed as drug-delivery vehicles. Sun et al. studied a model for 1D materials called anodic alumina nanotubes (AANTs), which induce intracellular autophagy when entering cells and degrade through the endolysosomal pathway. This lysosomal degradation process is blocked by applying autophagy inhibitors [91].

Photosensitive nanosized metal–organic frameworks (nanoMOFs) have been designed for photodynamic therapy (PDT). As autophagy is increased through the production of cytotoxic reactive oxygen species (ROS) during PDT and thus limits the efficacy of treatment, Sun et al. fabricated a chloroquine phosphate (CQ)-loaded photosensitive nanoMOF coated by heparin, which enhanced the tumor accumulation of nanophotosensitizers and blocked the self-protective autophagy into cancer cells. Since encapsulated CQ can alkalize autolysosomes and inhibit the post-autophagic process in previously irritated cancer cells via PDT, its therapeutic effect will be remarkable [92].

Treatment during pregnancy has always been a challenging issue as fetal safety should be considered. Nanoparticles can assist pregnant women with targeted drug delivery without undesirable off-target effects. However, the extent of fetal exposure to NPs crossing the placenta is not clear [93]. Based on the microfluidic principles, the placenta-on-a-chip technology was established and experimented in vivo, ex vivo, and in vitro to achieve drug delivery. By harnessing microfluidic principles, this technology can be used to adjust minute fluid volumes through narrow channels, guided by controlled forces [94].

#### Delivery of Autophagy-Regulating Drugs

Alzheimer’s disease (AD), a prevalent neurodegenerative disorder, is characterized by the accumulation of Aβ peptide aggregates within specific brain regions. Autophagy has a vital role in the clearance of proteins implicated in AD pathogenesis [95]. Autophagy dysregulation is a significant cause of AD, with extensive research revealing a close interplay between impaired autophagy and protein aggregation in the disease context [95,96,97]. Consequently, modulating autophagy presents a promising avenue for therapeutic intervention in AD [9]. The elimination of ATG7 and ATG5 in adult animals triggers neurodegeneration and mortality primarily due to the buildup of ubiquitinated protein complexes. Conversely, boosting autophagy flux counteracts protein accumulation, leading to cellular and organismal well-being. The PI3K/Akt/mTOR complex, which inhibits autophagy via ULK1, is activated by reactive oxygen species (ROS). Besides mTORC1, which activates or inhibits autophagy proteins, other kinases such as ERK or MEK act as triggers of autophagy onset, leading to cell survival. PTEN is a protein that has a role in the balance between PI3K and ERK pathways to mTOR modulation or autophagic gene transcription [98]. Two approaches for autophagy modulation that have been considered include small molecule therapeutics (e.g., berberine, sirolimus, or trehalose) and genetic intervention (i.e., gene therapy with TFEB or BECN1) [99]. The administration of small molecules modulates the PI3K/BECN1 autophagic pathway, resulting in the clearance of tau aggregates [100]. Different compounds have been proven to modulate mTORC1 activity and the autophagic process. Rapamycin, for instance, directly impacts mTORC1, making it a prominent modulator [98].

Autophagy impairment is involved in the dopaminergic neurodegeneration in Parkinson’s disease (PD) as well [101]. In the context of PD, lysosomal impairment is considered a major pathologic factor. Bourdenx et al. examined poly (DL-lactide-co-glycolide) (PLGA) acidic nanoparticles (aNPs), which have been approved by the FDA [102], on genetic cellular models of PD. The results showed that PLGA-aNPs can reacidify damaged lysosomes and restitute lysosomal function [103]. Zhu et al. delved into the potential of Apelin-36 as a therapeutic approach for PD. The results of their study conducted on a PD model in vitro showed that Apelin-36 acts as a cytoprotective agent through the PI3K/Akt/mTOR autophagy pathway [101]. Drugs that modulate PTEN activity, PPARα, and mGluR5 are candidates for the treatment of neurodegenerative diseases [98].

Small molecules that modulate autophagy in AD include Lu AE58054 (idalopirdine) [104], SB-742457 [105], nicotinamide [106], resveratrol [107], lithium [108], latrepirdine [109], and metformin [110], and SAGE217 is known to modulate autophagy in PD [111], all of which are under investigation in clinical trials, with potential future therapeutic objectives.

Hypoxia-induced ROS overproduction in bone diseases is a major challenge in bone regeneration. To restore the hypoxic condition of the bone microenvironment, Sun et al. designed hydrogels that acted as ROS scavengers and oxygen generators (CPP-L/GelMA). These GelMA hydrogels contained catalase and nanoparticles (PFC@PLGA/PPS) coloaded liposome (CCP-L) releasing oxygen in response to ROS. By releasing catalase and degrading hydrogen peroxide, oxygen was generated, leading to osteogenesis and angiogenesis as well as the inhibition of osteoclastogenesis in a mice skull defect model involving the Nrf2-BMAL1 autophagy pathway [112].

Silicate nanoparticles (C2S NPs) induce osteoblastic differentiation in BMSCs and promote the expression of LC3 and Beclin. C2S NPs involve autophagy activation by suppressing mTOR, thus assisting ULK1 expression and the activation of the WNT/β-catenin pathway. This subsequently facilitates osteoblast differentiation and biomineralization [113].

Nanodrugs can be used for the regulation of the immune microenvironment for bone regeneration. As an autophagy inducer, rapamycin triggers bone regeneration. However, due to its low bioavailability and high-dose-mediated cytotoxicity, some alterations have been achieved in studies on the use of rapamycin for clinical applications. In this regard, rapamycin-loaded virus-like hollow silica nanoparticles (R@HSNs) have been developed. R@HSNs can be translocated to lysosomes and act as a trigger of macrophage autophagy, leading to bone regeneration through the osteogenic differentiation of mBMSCs [114].

Autophagy regulatory drugs in cancer treatment include several agents that either inhibit the cytoprotective role of autophagy or induce autophagy in apoptosis-resistant cells [115].

There are some known autophagy-inhibiting molecules, categorized into four groups based on their mechanism of action: (1) Autophagosome formation inhibitors target the early stages of autophagy, preventing the formation of autophagosomes. Examples include 3-methyladenine (3-MA), wortmannin, LY294002, SAR405, and viridiol. (2) Inhibitors of lysosomal acidification disrupt the proper acidification of lysosomes, which is essential for the degradation of autophagic cargo. This leads to the inhibition of autophagic flux. Examples include chloroquine (CQ), hydroxychloroquine (HCQ), Lys0569, and monensin. (3) Inhibitors of autophagosome–lysosome fusion prevent the fusion of autophagosomes and lysosomes, thereby blocking the degradation of autophagic cargo. Examples include bafilomycin and concanamycin. (4) Autophagy-related gene silencers of transcription act at the genetic level to silence the expression of autophagy-related genes, leading to a decrease in autophagic activity. This can be achieved through siRNA- or miRNA-mediated silencing strategies [116,117,118,119,120,121].

As anticancer drugs, CQ and HCQ have shown antiautophagic activities via autophagic flux inhibition, lysosomal pH enhancement, the activation of p53 and TLR9/nuclear factor kappa B (NF-κB) signaling pathways, or the inhibition of CXCL12/CXCR4 signaling pathway [122,123].

Berberine is a drug that has been recently used for the prevention and treatment of gastric cancer. It acts through different mechanisms such as the regulation of inflammatory cytokines, the regulation of macrophage polarization, and the induction of autophagy [124].

Autophagy regulates the inflammasome and long-term inflammation, resulting in early cancer development [125]. Stressors increase autophagy activation, and then Beclin-1, a crucial autophagy gene, is upregulated in many tumor cells, including colorectal, gastric, liver, breast, and cervical cancer cells. Studies on targeting autophagy in breast cancer have shown that autophagy inhibition reduces drug resistance and improves drug response [126,127]. On the other hand, preclinical data showed that targeting autophagy may play a role in tumor suppression and cancer cell death as autophagosomes and autolysosomes were found to accumulate in dying cells, while apoptosis was not activated [128]. In advanced cancers, both autophagy activation and inhibition have been applied as therapeutic strategies [129].

The role of autophagy in cancer is complex and is known to be a double-edged sword, as revealed in numerous studies. On the one hand, it has tumorigenic and prometastatic roles depending on the context and stage of cancer and also the interaction between autophagy and Wnt signaling pathways [4,130]. For instance, it has been shown that autophagy is involved in melanoma metastasis, and autophagy inhibition can improve the sensitivity of melanoma cells to chemotherapy [131]. On the other hand, it acts as an anticancer factor [4]. For example, flavopiridol (FP) has shown antitumoral effects in breast cancer via autophagy involvement, and if autophagy is inhibited, tumor cells will survive [132]. Resveratrol is another agent that induces autophagy in several cancers through an increase in p62 degradation and mTOR and Nrf2 inhibition, leading to preventive and therapeutic effects [133].

Indeed, autophagy leads to antitumoral effects during the early stages of autophagy because of its protective role against metabolic, oxidative, and inflammatory stress [134]. In the early stages of tumorigenesis, autophagy can preserve genome stability; inhibit the accumulation of oncogenic p62 protein aggregates; and prevent tumor initiation, proliferation, invasion, and metastasis, leading to the activation of tumor suppressive mechanisms [127]. However, in the later stages, autophagy acts as a cellular protective mechanism that can maintain mitochondria and facilitate their survival, reduce DNA damage, and increase the survival and resistance of cancer cells against stressors such as hypoxia and chemotherapy, leading to tumor development and resistance to therapeutic drugs [135].

Various types of nanomaterials are being explored for their potential to modulate autophagy in cancer therapy. Gold nanoparticles, liposomes, and DNA-based nanostructures are candidates that exhibit high biocompatibility, structural diversity, low toxicity, and the ability to penetrate cell membranes without the need for transfection [136]. Ovarian cancer (OC) has shown resistance to cisplatin, used in conventional therapy. Chemotherapeutic nanomaterials such as polyethylenimine (PEI)-caged platinum nanoclusters (Pt NCs) on cisplatin-resistant OC activate autophagy via the PI3K/AKT/mTOR pathway inhibition. Pt NCs are among the promising nanomaterial-based drugs for OC treatment [137]. Graphene-based nanomaterials (GNMs) are another class of materials that have shown the potential to exert anticancer effects by influencing autophagy. They can induce autophagy or suppress the autophagic flux, ultimately leading to tumor cell death or the modulation of immune responses against tumors [138]. These innovative approaches utilizing nanomaterials demonstrate the potential for targeting autophagy as a strategy for improving cancer therapy outcomes.

### 2.4. Biomaterials, Autophagy, and Neurodegenerative Diseases

Neurodegenerative diseases are progressive disorders that are prevalent among older people. Neuroinflammation as a defensive response against various pathogenic stimuli has detrimental effects on host tissue, and chronic inflammation has been proven to contribute to various neurodegenerative disorders, including Alzheimer’s disease (AD), Parkinson’s disease (PD), Huntington’s disease, and amyotrophic lateral sclerosis (ALS) [25]. Epidemiological studies have revealed a relationship between exposure to nanoparticles and neurological diseases such as Alzheimer’s disease and Parkinson’s disease. Autophagy impairment in AD is responsible for disease progression in various ways [139,140]. In a previous study by Stern et al., increased levels of autophagic vacuoles were observed when cells were exposed to some nanomaterials, indicating that there is an interaction between the autophagy pathway and nanomaterials [141]. Amyotrophic lateral sclerosis (ALS) is a neurodegenerative disease with an autophagy impairment mechanism. Stem cell therapies, gene therapies, and newly developed biomaterials present promising tools for alleviating neurodegeneration, which might curb disease progression. Dysfunctional organelle clearance and the elimination of abnormal protein levels in cells through autophagy are essential to neuron homeostasis. Then, autophagy-targeting therapies can be effective in ALS treatment. Nanotechnology-based strategies have improved therapeutic approaches, including the delivery of drugs, genes, and antisense oligonucleotides (ASOs) to the CNS and boosting the effectiveness of stem cell therapies [142,143]. Riluzole carried by solid lipid nanoparticles causes higher levels of the drug in the brains of rats [144]. It is also revealed that encapsulated riluzole in Tween-80-coated, chitosan-conjugated N-isopropylacrylamide nanoparticles cross the blood–brain barrier and then decreases inflammatory agent expression and increases glutathione concentration, leading to neuroprotection [145]. Liposomal nanoparticles reduce drug resistance, block efflux transporters, and improve drug uptake [146].

In Alzheimer’s disease, another neurodegenerative disorder, elevated levels of intracellular amyloid-β (Aβ) have neurotoxicity effects. Autophagy dysfunction is one of the causes of pathogenesis, and its activation can help intracellular amyloid-β (Aβ) elimination. Biomaterials have beneficial effects in this area. Liu et al. synthesized an autophagy inducer using quercetin (Qu)-modified polysorbate 80 (P-80)-coated AuPd core–shell structure and applied it on SH-SY5Y cells. It was found to activate autophagy, fuse autophagosomes and lysosomes, accelerate the elimination of Aβ, and preserve SH-SY5Y cells from the cytotoxicity effects of Aβ. Due to its biocompatibility and high blood–brain barrier (BBB) permeability, concave cubic Qu@P-80@AuPd can serve as an autophagy activator in AD treatment [147]. Table 4 shows the most important feature of nanomaterials that could influence autophagy in neurodegenerative diseases.

### 2.5. Biomaterials, Autophagy, and Angiogenesis

Vascularization is an essential therapeutic strategy for tissue regeneration in wound healing, cardiovascular disease treatment, and tissue engineering. Nanomaterials have unique structural properties that promote angiogenesis through various mechanisms. The directional migration of endothelial cells, the proliferation of adjacent cells, and the formation of tubules occur during angiogenesis after stimulation by proangiogenic factors. Nanomaterials participate in all the abovementioned stages [150]. The activity of osteoprogenitor cells in bone tissue engineering, including migration, differentiation, and bone formation, requires oxygen and nutrient supply, in addition to the interaction of endothelial cells and osteocytes, leading to cell survival and bioengineered bone integration with the host tissue [151]. Efficient angiogenesis such as the growth and adhesion of human umbilical vein endothelial cells (HUVECs) depends on the nanomaterial composition that improves the mechanical properties and surface hydrophilicity of bone scaffolds [152]. Nanoscaffolds also act as carriers of proangiogenic molecules or proteins, including deferoxamine, adrenomedullin, and VEGF [153,154]. Using different micro-/nanostructured surfaces on HA scaffolds in copper ion solution (Cu^2+^) showed that the use of copper as a trace element upregulates the VEGF expression and promotes endothelial cell proliferation [155]. The shape and size of nanoparticles are important characteristics in their interaction with endothelial cells and angiogenesis. In vivo and in vitro studies have shown that spherical neodymium has the best biocompatibility to cell proliferation. Nanoparticle morphologies cause cellular uptake and autophagy activation, which makes hydroxyapatite nanospheres a great angiogenic biomaterial [70]. Autophagy promotes angiogenesis in inflammatory conditions [156,157]. Transcription factor EB (TFEB) is a key regulator of autophagy that promotes endothelial cell proliferation and autophagic flux and regulates the G1-S transition, leading to angiogenesis [158]. Nanomaterials may play a role in protective autophagy. For instance, nuclear translocation of TFEB is induced by silver nanoparticles, leading to the expression of autophagy genes and cell survival [159]. Experiments showed that the protective effects of autophagy cause the proliferation and differentiation of endothelial progenitor cells into endothelial cells [160]. In another study, a type of sprayable adhesive hydrogel was fabricated and coloaded with a complex of zinc and metformin (ZnMet). The fabricated hydrogel promoted the healing of traumatic skin defects and injured wounds via the inhibition of ROS production through the activation of autophagy. In addition, it could improve cell proliferation, collagen formation, and angiogenesis [161]. Bioinspired hydroxyapatite nanoparticles were fabricated in another study and were applied in the structure of composite scaffolds to evaluate their effects on the promotion of vascularized bone regeneration. The results confirmed morphology-dependent vascularization and autophagy-mediated osteogenesis and cell proliferation, so spherical nanoparticles were more effective in these cases [70]. Table 5 illustrates some of the features of nanomaterials that can promote angiogenesis through autophagy mechanism involvement.

## 3. Nanomaterial-Mediated Autophagy Risks

Despite all its health benefits, dual effects of nanomaterial (NM)-mediated autophagy have been reported in both in vivo and in vitro studies [37]. Oxidative stress, inflammation, cell apoptosis, and other unknown mechanisms occur as a result of NM-driven toxicity [165,166]. Some studies have reported that autophagy can develop cancer [167], autoimmune diseases [168], and cardiovascular diseases [169]. The reported hepatotoxicity [170,171], nephrotoxicity [159,172,173], pulmonary toxicity [174,175], neurotoxicity [176,177,178], and cardiovascular toxicity [179,180] are concerning aspects related to the biosafety of NM applications. It seems that how to use of NMs for the regulation of the autophagic process determines the value of NMs in clinical treatment [37].

The consideration of concentration-dependent toxicity limits is crucial when working with targeted nanoparticles, as it impacts nanoparticles’ safety and effectiveness. Using higher concentrations of nanoparticles might lead to unwanted toxic effects that can offset their therapeutic benefits. For instance, quartz nanoparticles (QNPs) increase ROS production and lipid peroxidation and induce DNA damage on A549 cells in a concentration- and time-dependent manner [181]. Some strategies should be employed to reduce the toxicity of nanoparticles. For example, the addition of N-acetylcysteine (NAC) to silver nanoparticles (AgNPs) results in a protective effect on AgNP-induced hepatotoxicity. Also, Nrf2 siRNA transfection inhibits the concentration-dependent increase in apoptosis induced by AgNPs in HepG2 cells and L02 cells [182]. Other samples related to the toxicity mechanisms of nanomaterials are summarized in Table 6.

## 4. Conclusions and Future Perspectives

The study of autophagy and its interactions with biomaterials holds great promise for various applications in the fields of diagnosis, treatment, tissue engineering, and targeted drug delivery. Nanomaterials, with their unique physicochemical properties, have emerged as key components in medical strategies in diverse areas such as osteogenesis, angiogenesis, neurodegenerative disease treatment, and cancer therapy. It is important to consider both the advantages and disadvantages of nanomaterial-mediated autophagy. This mini-review shed light on the effects of autophagy following the use of biomaterials and discussed the possible related mechanisms.

An exciting avenue for future biomaterial development lies in fabricating advanced nanomaterials with tailored properties for precise autophagy modulation. Nanomaterials, with their unique physicochemical characteristics, can be designed to interact with cellular processes and precisely regulate autophagy pathways. By engineering the properties of nanomaterials, such as size, shape, surface chemistry, and mechanical properties, researchers can exert fine control over autophagy modulation. Moreover, the incorporation of nanomaterials into implantable devices and scaffolds used in tissue engineering opens new possibilities for the control of autophagy in specific cell types or tissues [199,200]. Biomaterial-based platforms can be engineered to provide spatial and temporal control over autophagic processes, promoting tissue regeneration or selectively targeting autophagy in diseased cells. These advances pave the way for personalized medicine approaches, tailoring autophagy modulation strategies to individual patient needs [201]. In the field of cancer therapy, biomaterials offer exciting prospects for autophagy modulation. Nanoparticle-based drug-delivery systems can be designed to trigger autophagy in cancer cells, enhancing their sensitivity to chemotherapy or promoting their death through autophagic cell death [202]. Conversely, biomaterials can be engineered to inhibit autophagy in tumor cells, preventing their survival and promoting their susceptibility to traditional therapeutic agents. This multifaceted approach highlights the potential of biomaterials in overcoming drug resistance and improving cancer treatment outcomes [138]. Furthermore, the integration of biomaterials with advanced technologies, such as microfluidics and biofabrication techniques, presents new avenues for studying autophagy and developing innovative diagnostic tools. Microfluidic devices can mimic the tumor microenvironment and enable precise control over autophagic processes, providing insights into the interplay between biomaterials, autophagy, and disease progression [203]. Biofabrication techniques, such as 3D bioprinting, allow complex tissue models to be built with controlled autophagy modulation, facilitating the study of autophagy-related diseases and accelerating the development of targeted therapies [204].

While the future of biomaterials in modulating autophagy holds tremendous promise, further research is needed to delve deeper into the intricate relationship between autophagy and biomaterials. Investigations should aim to elucidate the specific mechanisms through which biomaterials modulate autophagy and determine the optimal conditions for achieving desired outcomes. Additionally, studies should focus on the potential adverse effects and risks associated with nanomaterial-mediated autophagy. It is crucial to thoroughly assess the safety and long-term effects of these interventions to ensure their clinical feasibility and translation into practical applications. Moreover, exploring the therapeutic potential of autophagy modulation through biomaterials opens up exciting possibilities. The ability to fine-tune autophagic processes using biomaterial-based interventions may pave the way for novel therapeutic strategies in the treatment of autophagy-related diseases. Understanding the intricate interplay between autophagy and biomaterials will not only enhance our knowledge of fundamental cellular processes but also offer new avenues for the development of innovative diagnostic and therapeutic approaches. Apart from these, the robust characterization and thorough assessment of the biocompatibility and long-term effects of biomaterials are essential to ensure their safe and effective application in clinical settings.

In conclusion, the future of biomaterials in modulating autophagy is filled with exciting possibilities. From tailored nanomaterials to advanced tissue engineering platforms, these biomaterial-based approaches offer unprecedented control over autophagic processes, enabling personalized and targeted interventions in various biomedical applications. Continued research and interdisciplinary collaborations will drive innovation, deepen our understanding of autophagy mechanisms, and pave the way for the development of next-generation biomaterials with enhanced autophagy modulation capabilities, ultimately improving patient outcomes and advancing the field of regenerative medicine and therapeutics.

## Figures and Tables

**Table 1:** Effects of different features of nanomaterials on autophagy [38,39].

Properties of Nanomaterial	Effect on Autophagy
Dispersity	The aggregation of nanomaterials enhances autophagy.
Size	Smaller particles could activate autophagy more than larger ones due to their higher surface-to-volume ratio. They can activate autophagosomes and induce mitochondrial damage via the PI3K/Akt/mTOR signaling pathway. However, autophagy induction is different based on the type of nanoparticles.
Charge	All different charges of nanoparticles (anionic, cationic, and neutral particles) activate autophagy; however, neutral and anionic particles can improve the clearance of autophagic cargo, while cationic particles induce the accumulation of autophagosomes.
Surface chemistry	Bare nanomaterials can enhance autophagy activation; however, surface functionalization with polymeric materials can reduce this toxic effect.
Degradability	Non-degradable nanoparticles can improve the induction of autophagy.

In the following sections, we summarize some of the applications of bionanomaterials and their characteristic features in regulating autophagy.

**Table 2:** Nanomaterial characteristics that influence autophagy in osteogenesis.

Property of Nanomaterials	Type of Nanomaterials	Impact on Autophagy	Ref.
Structure	Hydroxyapatite groove structure	−/Decrease in LC3-2 expression	[67]
	SDF-1α-loaded silk fibroin scaffolds	+/Expression of LC3 and Atg5	[65]
	Strontium (Sr)-doped 45S5 bioglass (Sr/45S5)	+/Activation of the Akt/mTOR signaling pathway	[66]
Size	Titanium (Ti) particle	+/Expression of LC3	[71]
	Nanosized Alumina (Al)	−/Expression of LC3	[71]
	Gold nanoparticles	+/Upregulation of microtubule-associated protein light chain 3 (MAP1LC3) and downregulation of sequestosome 1/p62	[63]
Surface topography	Nanotube (NT)	+/Enhanced mTOR-independent autophagy	[44]
	Nanoporous anodic alumina	+/Activation of LC3A/B, Beclin-1, Atg3, Atg7, and P62	[44]
	Titanium–spherical silica nanoparticles	+/Improved formation of autophagosome	[57]

+ (plus): Activation of autophagy; − (minus): inhibition of autophagy; NM: not mentioned.

**Table 3:** Nanomaterial characteristics that influence autophagy in cancer cells.

Specifications	Nanomaterials	Impact on Autophagy	Ref.
Composition	Zinc nanofibers	+/Autophagosome and autolysosome formation	[75]
	CQ-containing cationic copolymer (PD3CQ1)	−/Increased the unfused autophagosomes	[82]
	Iron–gold core–shell nanoparticles	+/Mitochondria-mediated autophagy	[86]
Charge	Peanut-shaped gold nanoparticles	+/Disturbing mitochondrial function by enhancing ROS and JNK signaling pathway	[87]
Size	Ag nanoparticles	+/Smaller sizes are more effective in inducing LC3B protein production	[88]

+ (plus): Activation of autophagy, − (minus): inhibition of autophagy.

**Table 4:** Nanomaterial characteristics that influence autophagy in neurodegenerative diseases.

Specification	Nanomaterials	Impact on Autophagy	Ref.
Composition	Quercetin-modified gold–palladium nanoparticles	+/Promote the fusion of autophagosomes and lysosomes, clearing Aβ and decreasing Aβ-mediated cytotoxicity, high BBB permeability	[147]
	Graphene oxide	+/Activation of PtdIns3K and MEK/ERK1/2 signaling pathways	[148]
	SiO_2_ nanoparticles	+/Inhibition of PI3K-Akt-mTOR signaling	[149]

+ (plus): Activation of autophagy.

**Table 5:** Nanomaterial characteristics that influence autophagy leading to angiogenesis.

Specification	Nanomaterials	Impact on Autophagy	Ref.
Height (13 and 35 nm)	Poly (styrene) nanohills	+/Greater adhesion	[162]
Shape (spherical)	Neodymium nanoparticles	+/Cell proliferation	[163]
Shape	Nanohydroxyapatite	+/Better angiogenic potential	[164]

+: Activation of autophagy.

**Table 6:** Different toxicity effects of biomaterials.

Nanoparticles	Toxicity Mechanism	Ref.
ZnO NPs	Increased production of intracellular reactive oxygen species (ROS), increased levels of LC3A, and finally autophagic death of immune cells	[183]
	Nephrotoxicity: cyto- and genotoxicity in the epithelial cells resulted from ROS and HIF-1α signaling pathway	[184]
	Oxidative stress in macrophages, cytotoxic, genotoxic, clastogenic, actin depolymerization	[185]
	Pulmonary inflammation: increases in coagulation factor VIII	[186]
TiO_2_	Hyperplasia and inflammation in a dose-dependent manner	[187]
	Endoplasmic reticulum (ER) and mitochondria disruption	[188]
SiNPs	Proinflammatory responses, oxidative stress, and autophagy	[189]
	Liver toxicity and thrombocytopenia	[190]
Graphene oxide nanoparticles	In vitro: dose-dependent cytotoxicity, apoptosis, DNA damage, released LDH, increased MDA and ROS generation, decreased SOD, reduction in cell viability	[191]
Rapamycin	Cytotoxicity	[114]
Cd-based QDs	Oxidative stress resulting in mitochondrial or DNA damage	[192]
Chitosan-based nanomaterials	Toxicity in different organs	[193]
	Teratogen	[194]
Gold nanoparticles (AuNPs) in BALB/c mice	Apoptosis and inflammation of liver tissue	[195]
PEG-coated AuNPs in Rat	ROS-induced cytotoxicity that is size-dependent	[196]
Silver nanoparticles (AgNPs)	ROS-mediated stress, resulting from the tissue-wide accumulation of ROS	[197]
Superparamagnetic iron oxide (SPIO) NPs	Cell membrane damage	[198]
Carbon nanotubes (MWCNTs)	In pulmonary exposure, an increase in blood neutrophils, an increase in coagulation factor VIII, and alveolar inflammation	[186]

## Data Availability

No new data was created or analyzed in this study. Data sharing is not applicable to this article.
